# COVID-19 and helminth infection: Beyond the Th1/Th2 paradigm

**DOI:** 10.1371/journal.pntd.0009402

**Published:** 2021-05-24

**Authors:** Alberto E. Paniz-Mondolfi, Juan David Ramírez, Lourdes A. Delgado-Noguera, Alfonso J. Rodriguez-Morales, Emilia M. Sordillo

**Affiliations:** 1 Department of Pathology, Molecular and Cell-Based Medicine, Icahn School of Medicine at Mount Sinai, New York, New York, United States of America; 2 Centro de Investigaciones en Microbiología y Biotecnología-UR (CIMBIUR), Facultad de Ciencias Naturales, Universidad del Rosario, Bogotá, Colombia; 3 Incubadora Venezolana de la Ciencia, Instituto de Investigaciones Biomédicas IDB, Barquisimeto, Venezuela; 4 Grupo de Investigación Biomedicina, Faculty of Medicine, Fundación Universitaria Autónoma de las Américas, Sede Pereira, Pereira, Risaralda, Colombia; University of Hong Kong, HONG KONG

In a recent commentary, Bradbury and colleagues raise concerns regarding the potential influence of preexisting helminth infections on Coronavirus Disease 2019 (COVID-19) disease severity in helminth-endemic regions [1]. They suggest that modulation of the immune response due to boosted Th2-like cytokine responses in helminth-infected persons could regulate the intensity of the inflammatory response to COVID-19, which has been attributed to an uncontrolled Th1 proinflammatory cytokine response that can be linked directly to disease severity. However, the authors also point out that in contrast to infections with SARS and some other viruses, the response in COVID-19 includes elevation of IL-4 and IL-10, considered to be type 2 cytokines [1].

In response, Hays and colleagues [2] offer the view that preexisting helminth infections, by decreasing the likelihood of metabolic syndrome and type 2 diabetes mellitus, may reduce the risk of cytokine storm and severe COVID-19 [2]. Along the same lines, Siles-Lucas and colleagues [3] provide hypothetical insights regarding potential modulating effects by which specific helminth-derived molecules might modulate COVID-19 pathology. However, the authors also cautiously note that animal studies on parasite-viral coinfection remain equivocal, and likewise highlight the need for further studies to directly assess the potential impact of helminth infections on COVID-19 severity.

An important caveat for consideration is that caution should be exercised when interpreting data from endemic areas due to possible lags in reporting. Evidence from some prospective surveillance studies in Africa suggest that the number of COVID-19 cases is underreported [4]. Furthermore, emerging epidemiologic data from the Amazon basin in South America do not support the suggestion of decreased illness severity in helminth-endemic regions. This region has been heavily impacted by the expanding Severe Acute Respiratory Syndrome Coronavirus 2 (SARS-CoV-2) pandemic wave [5]. Among the Xavante, Hüpda, Kaingáng, and other Amerindian peoples of the Brazilian Amazon, the prevalence of soil-transmitted helminths, predominantly *Ascaris lumbricoides* and *Ancylostoma* sp., reaches 45% to 95% [6], but within Brazil, Amazonas has been the jurisdiction most affected by SARS-CoV-2 thus far, with a death rate 250% higher than in the rest of Brazil [5]. The Colombian Amazon, an area in which the prevalence of intestinal parasitosis reaches 70.5%, similarly has been severely impacted by SARS-CoV-2 [7]. In Venezuela, parasitosis with soil-transmitted helminths in rural communities has been estimated to affect at least 65% [8]. This population has been severely affected by the ongoing pandemic, although data regarding mortality in Venezuela are limited [5]. Thus, in these South American populations, the high prevalence of intestinal parasitosis with soil-transmitted helminths does not appear to have provided a benefit in terms of SARS-CoV-2-related morbidity or mortality.

Conversely, data from Lucas and colleagues regarding patterns of immune response to SARS-CoV-2 infection may help clarify why activation of the canonical helminth-driven Th2 phenotype could instead be deleterious [9]. In their study, 3 distinct immune profiles were observed in response to SARS-CoV-2 infection and could be correlated with disease severity. Patients with severe COVID-19 displayed a number of type 2 phenotype effectors including IL-5, IL-13, immunoglobulin E, and eosinophils [9]. Similar findings have been reported by Oja and colleagues in severely ill COVID-19 patients [10]. Additionally, other factors may be at play beyond the fixed dogma of Th1/Th2 responses. Inherited variation in innate immune response genes to viruses as well as cytokine gene polymorphisms also may contribute to the heterogeneous clinical outcomes seen globally among different geographical areas and communities as well as to the disparate clinical outcomes among patients. For example, mutations in the human leukocyte antigen (HLA-DQA1) has demonstrated to play a role on increasing susceptibility to smallpox among Native American populations. The influence of the human virome/microbiome on the immune response to new infections may also be an important contributor to response to SARS-CoV-2 [11]. In fact, a recent study by Yatsunenko and colleagues [12] has revealed differences in bacterial assemblages and functional gene repertoires between Amerindians in the Venezuelan Amazon when compared to US residents.

Lastly, it is essential to remember the manifold negative effects of intestinal parasitosis. The hookworms *Ancylostoma duodenale* and *Necator americanus* cause blood loss, anemia, pica, and wasting. Roundworms can also cause malabsorption, compete for nutrients, or cause intestinal or biliary tract obstruction, or in some cases, dysentery or diarrhea [13]. In regions where undernutrition rather than overnutrition is a dominating concern, nutritional and metabolic compromise may present a greater hazard in persons at risk for SARS-CoV-2 infection.
